# Antitumor Effect of Albendazole on Cutaneous Squamous Cell Carcinoma (SCC) Cells

**DOI:** 10.1155/2019/3689517

**Published:** 2019-06-09

**Authors:** Qing-Ling Zhang, De-De Lian, Ming Ji Zhu, Xue Mei Li, Jae Kyung Lee, Tae-Jin Yoon, Jeung-Hoon Lee, Ri-Hua Jiang, Chang Deok Kim

**Affiliations:** ^1^Department of Dermatology, China-Japan Union Hospital of Jilin University, Changchun, Jilin, China; ^2^Department of Dermatology, School of Medicine, Chungnam National University, Daejeon, Republic of Korea; ^3^Department of Intensive Care Unit, China-Japan Union Hospital of Jilin University, Changchun, Jilin, China; ^4^Department of Medical Science, School of Medicine, Chungnam National University, Daejeon, Republic of Korea; ^5^Department of Dermatology and Institute of Health Sciences, School of Medicine, Gyeongsang National University & Hospital, Jinju, Republic of Korea; ^6^Skin Med Company, Daejeon, Republic of Korea

## Abstract

Drug repurposing and/or repositioning is an alternative method to develop new treatment for certain diseases. Albendazole was originally developed as an anthelmintic medication, and it has been used to treat a variety of parasitic infestations. In this study, we investigated the antitumor effect of albendazole and putative action mechanism. Results showed that albendazole dramatically decreased the cell viability of SCC cell lines (SCC12 and SCC13 cells). Albendazole increased apoptosis-related signals, including cleaved caspase-3 and PARP-1 in a dose-dependent fashion. The mechanistic study showed that albendazole induced endoplasmic reticulum (ER) stress, evidenced by increase of CHOP, ATF-4, caspase-4, and caspase-12. Pretreatment with ER stress inhibitor 4-PBA attenuated albendazole-induced apoptosis of SCC cells. In addition, albendazole decreased the colony-forming ability of SCC cells, together with inhibition of Wnt/*β*-catenin signaling. These results indicate that albendazole shows an antitumor effect via regulation of ER stress and cancer stemness, suggesting that albendazole could be repositioned for cutaneous SCC treatment.

## 1. Introduction

Cutaneous squamous cell carcinoma (SCC) is the cancer originated from upper layers of skin epidermis. It is the second most common nonmelanoma skin cancer, influencing the quality of life considerably [1]. Chronic exposure to ultraviolet (UV) radiation is the most important cause for SCC [2, 3]. Other main risk factors include the duration of chronic inflammation and immunosuppressive condition [4]. At cellular level, a variety of regulators related to cell growth and differentiation, such as p53 and Wnt/*β*-catenin signaling, are implicated in the development of SCC [5, 6]. For treatment of SCC, various methods can be applied. Surgical excision is the first-line treatment for the relatively large SCC. For the small and low-risk lesions, electrodesiccation and curettage combined with photodynamic therapy and cryosurgery can be used. Other modalities include topical medications, radiotherapy, and systemic therapy [7]. Although many treatment methods are currently applied, there is still a demand for the development of new drugs that have high efficacy, low side effects, and easy applicability. One attractive approach to develop the candidate materials is the repurposing and/or repositioning of existing drugs [8]. We attempted to screen the antitumor materials using a commercially available drug library and found that albendazole had a potential to induce the apoptosis of cutaneous SCC cells.

**Figure 1:**
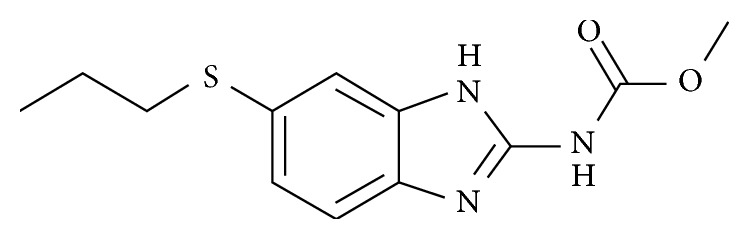
Structure of albendazole.

Albendazole (methyl N-(6-propylsulfanyl-1H-benzimidazol-2-yl)carbamate) is a benzimidazole derivative, which has been introduced in 1982 as an anthelmintic drug (Figure 1) [9]. The mechanism of albendazole as an anthelmintic medication depends on its inhibitory potential on microtubule formation and the ability of impairing glucose uptake, thereby depleting glycogen [10]. Subsequently, many studies indicate that albendazole has an antitumor activity. For example, albendazole inhibits growth of non-small cell lung cancer cells by suppressing hypoxia-inducible factor-1-*α* (HIF-1-*α*) activity and vascular endothelial growth factor (VEGF) expression [11]. In another example, albendazole inhibits the growth of metastatic melanoma and increases sensibility of cancer cells to radiation [12]. It has been also demonstrated that albendazole triggers the apoptosis of human leukemia cells via SIRT3/ROS/p38 MAPK/TTP axis-mediated TNF-*α* upregulation [13]. Although the antitumor ability has been recognized in other systems, the effects of albendazole on cutaneous SCC cells and the action mechanism remain to be investigated.

## 2. Materials and Methods

### 2.1. Cell Culture

SCC12 and SCC13 cells are the human squamous cell carcinoma line, established from SCCs of the facial epidermis [14]. Both the cells were maintained in Dulbecco's modified Eagle's medium (DMEM) supplemented with 5% fetal bovine serum (FBS) and antibiotics (Life Technologies Corporation, Grand Island, NY) at 37°C, 5% CO_2_ atmosphere. Cells were routinely passaged with 1:5 ratio when they grew to nearly confluent. Normal human epidermal keratinocytes were isolated from skin specimens obtained in accordance with the ethical committee approval process of Chungnam National University Hospital. Specimens were briefly sterilized in 70% ethanol, minced, and then treated with dispase overnight at 4°C. The epidermis was separated and placed in a solution containing 0.05% trypsin and 0.02% EDTA (Life Technologies Corporation) for 15 min at 37°C. After vigorous pipetting, cells were pelleted and resuspended in keratinocyte-serum free medium (K-SFM) supplemented with bovine pituitary extract and recombinant human epidermal growth factor (Life Technologies Corporation). Albendazole and 4-phenylbutyric acid (4-PBA) were purchased from Sigma-Aldrich (St. Louis, MO) and dissolved in dimethyl sulfoxide (DMSO).

### 2.2. Cell Viability Test

SCC cells were treated with albendazole for 24 h; then medium was replaced with fresh medium containing 0.5 mg/ml 3-(4,5-dimethyl-2-thiazolyl)-2,5-diphenyl-2H-tetrazolium bromide (MTT). Cells were incubated for an additional 4 h, and then formazan crystal was dissolved in DMSO. Cell viability was determined by measuring optical density at 570 nm using an ELISA reader.

### 2.3. TUNEL Staining

Apoptotic cells were identified using an in situ Apoptosis Detection Kit (Abcam, Cambridge, UK). After treatment with albendazole, cells were incubated with a terminal deoxynucleotidyl transferase dUTP nick-end labeling (TUNEL) reaction mixture for 2 h at 37°C. Apoptotic cells were visualized by incubation with diaminobenzidine tetrachloride solution.

### 2.4. Western Blot

Cells were lysed in PRO-PREP solution (Intron, Daejeon, Korea). Total protein was measured using BCA protein assay kit (Thermo Scientific, Rockford, IL). Samples were run on sodium dodecyl sulfate-polyacrylamide gel (SDS-PAGE) and transferred onto nitrocellulose membranes. After blocking with 5% skim milk, the membranes were incubated with primary antibodies. Blots were then incubated with peroxidase-conjugated secondary antibodies and visualized by enhanced chemiluminescence (Intron, Daejeon, Korea). The following primary antibodies were used: poly(ADP-ribose) polymerase-1 (PARP-1), activating transcription factor-4 (ATF-4), caspase-4, caspase-12, *β*-catenin, and *α*-tubulin (Santa Cruz Biotechnologies, Santa Cruz, CA); CCAAT/enhancer-binding protein homologous protein (CHOP) and caspase-3 (Cell Signaling Technology, Beverly, MA); and *β*-actin (Sigma-Aldrich, St. Louis, MO).

### 2.5. Colony-Forming Assay

Cells were treated with albendazole for 24 h and then trypsinized and counted using a hemocytometer. After cell counting, about 3,000 cells were suspended in DMEM supplemented with 5% FBS and seeded into 100 mm culture dishes. Cells were incubated for 2 weeks and then stained with crystal violet (Sigma-Aldrich, St. Louis, MO).

### 2.6. Luciferase Assay

Cells were grown at 60% confluency in 24-well culture plates and then transduced with TOPflash reporter adenovirus overnight [15]. Cells were replenished with fresh medium containing albendazole and incubated for a further 24 h. Cellular extracts were prepared using cell lysis buffer, and then luciferase activity was determined using Luciferase assay system (Promega, Madison, WI).

### 2.7. Statistical Analysis

Data were evaluated statistically by one-way ANOVA or Student's* t*-test using SPSS software v 22.0 (IBM, Seoul, Korea). Statistical significance was set at P<0.01.

## 3. Results

### 3.1. Albendazole Induces Apoptosis of Cutaneous SCC Cells

**Figure 2:**
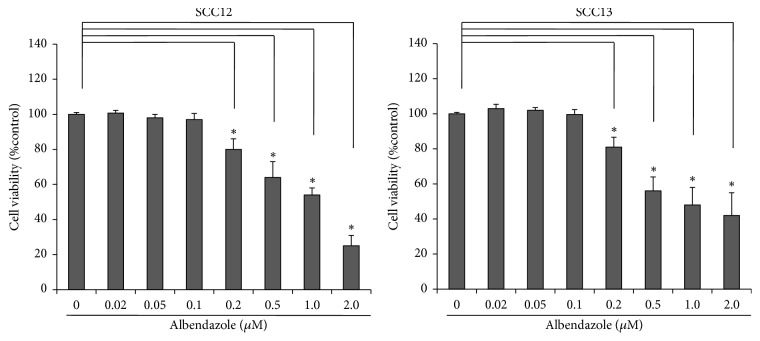
Cytotoxicity of albendazole in SCC cell lines. SCC12 and SCC13 cells were treated with albendazole at the indicated concentrations for 24 h. MTT assay was performed to determine cell viability. Albendazole induced the cell death at more than 0.2 *μ*M in both SCC12 and SCC13 cells. Data are expressed as percentage of control (0 mg/ml albendazole). The mean values ± SD are averages of triplicate measurements. *∗*P<0.01.

**Figure 3:**
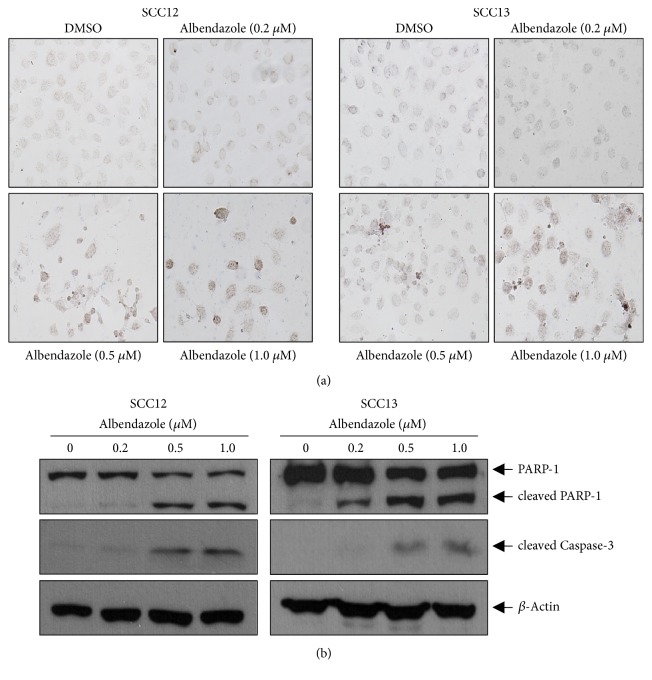
Albendazole induces apoptosis of SCC cells. (a) SCC12 and SCC13 were treated with albendazole at the indicated concentrations for 24 h. TUNEL staining was performed to detect apoptosis. Albendazole significantly increased the TUNEL-positive cells in a dose-dependent manner. (b) SCC12 and SCC13 were treated with albendazole for 24h. The protein levels of cleaved PARP-1 and caspase-3 were detected by Western blot. *β*-Actin was used for internal control. Cleavage of PARP-1 and caspase-3 increased significantly by albendazole treatment.

To investigate the effect on cell viability, we treated cutaneous SCC cell lines (SCC12 and SCC13) with albendazole and performed MTT assay. As a result, albendazole decreased the cell viability of SCC12 and SCC13 cells at the doses more than 0.2 *μ*M (Figure 2). To verify whether albendazole induces apoptosis of SCC12 and SCC13 cells, we carried out TUNEL staining. Compared to DMSO-treated control group, TUNEL-positive cells increased notably in the albendazole-treated SCC cell lines at the doses more than 0.5 *μ*M (Figure 3(a)). Consistent with these data, albendazole induced the cleavage of PARP-1 and caspase-3 in a dose-dependent manner. In SCC12 cells, albendazole induced the cleavage of PARP-1 at 0.5 *μ*M concentration, whereas the PARP-1 cleavage was evident at 0.2 *μ*M concentration in SCC13 cells (Figure 3(b)).

**Figure 4:**
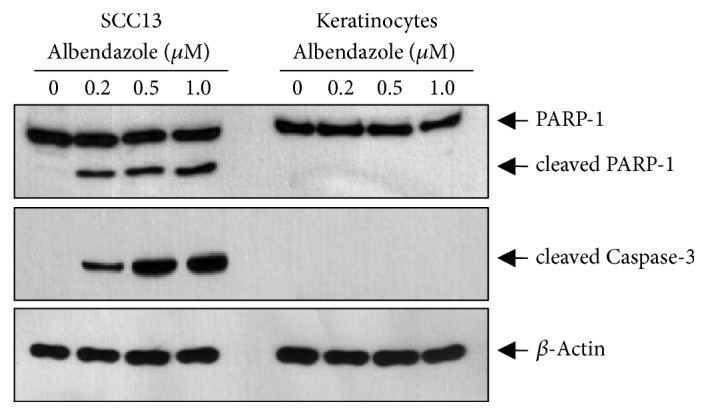
Effect of albendazole on apoptosis in SCC cells and keratinocytes. SCC13 cells and normal human epidermal keratinocytes were treated with albendazole at the indicated concentrations for 24 h. Cleavage of PARP-1 and caspase-3 increased significantly by albendazole treatment in SCC13 cells. However, albendazole did not induce cleavage of PARP-1 and caspase-3 in keratinocytes.

To investigate whether the effect of albendazole was specific for cancer cells, we performed the comparative test using normal human epidermal keratinocytes. Albendazole induced the cleavage of PARP-1 and caspase-3 in SCC13 cells at the doses more than 0.2 *μ*M, whereas it failed to induce the cleavage of PARP-1 and caspase-3 in keratinocytes even at 1.0 *μ*M concentration (Figure 4).

### 3.2. Albendazole-Induced Apoptosis Is Related to Endoplasmic Reticulum (ER) Stress

**Figure 5:**
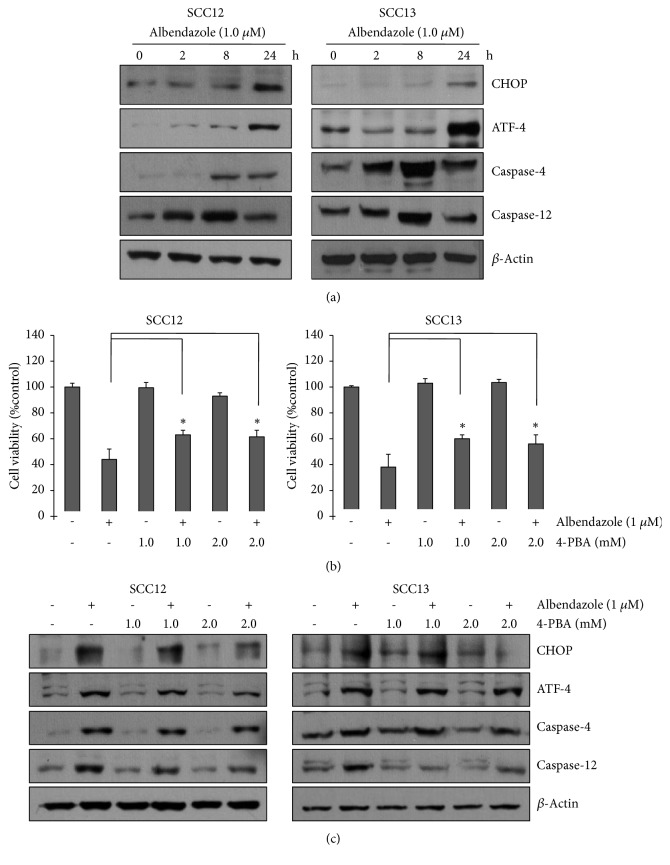
Albendazole-induced apoptosis is via the ER stress. (a) SCC12 and SCC13 cells were treated with 1 *μ*M of albendazole for the indicated time points. The ER stress markers were detected by Western blot. Albendazole induced the ER stress in a time-dependent manner. (b) SCC12 and SCC13 cells were pretreated with ER stress inhibitor 4-PBA at the indicated concentrations for 1 h and then treated with 1 *μ*M of albendazole for 24 h. Cell viability was measured by MTT assay. Inhibition of ER stress by 4-PBA attenuated albendazole-induced cell death. Data are expressed as percentage of control. The mean values ± SD are averages of triplicate measurements. *∗*P<0.01. (c) Cells were treated as in (b); then ER stress markers were detected by Western blot.

To delineate the putative mechanism underlying albendazole-induced apoptosis of SCC cells, we examined whether albendazole can trigger ER stress. After treatment with 1.0 *μ*M albendazole, the ER stress indicators such as CHOP and ATF-4 were markedly increased in a time-dependent manner (Figure 5(a)). If excess ER stress is not reversed appropriately, cellular functions deteriorate and often cells go to die by apoptotic process [16]. Thus, we next examined the effect of albendazole on caspase-4 and capase-12, which are implicated in ER stress-induced apoptosis [17]. As expected, albendazole increased the caspase-4 and caspase-12 in SCC12 and SCC13 cells (Figure 5(a)). These results suggest that albendazole-induced ER stress might be functionally involved in apoptosis of SCC12 and SCC13 cells. To verify this notion, we used an ER stress inhibitor 4-PBA [18]. SCC cells were pretreated with 4-PBA and then treated with albendazole. Cell viability test showed that albendazole-induced cell death was significantly inhibited by pretreatment with ER stress inhibitor (Figure 5(b)). Western blot revealed that 4-PBA pretreatment suppressed the albendazole-induced CHOP and caspase-12 activation, while ATF-4 and caspase-4 were not significantly affected (Figure 5(c)). These data support the notion that albendazole induces the apoptosis of SCC cells via triggering of ER stress.

**Figure 6:**
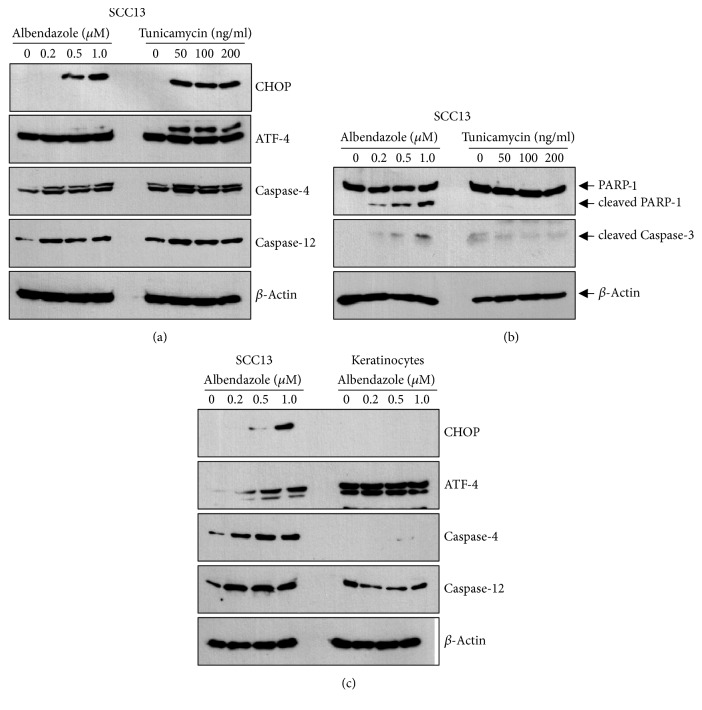
(a) SCC13 cells were treated with albendazole and tunicamycin at the indicated concentrations for 24 h. Albendazole and tunicamycin induced the ER stress in a dose-dependent manner. (b) Cleavage of PARP-1 and caspase-3 increased significantly by albendazole treatment in SCC13 cells. However, tunicamycin did not induce cleavage of PARP-1 and caspase-3. (c) SCC13 cells and normal human epidermal keratinocytes were treated with albendazole at the indicated concentrations for 24. Albendazole induced ER stress in SCC13 cells but not in keratinocytes.

We compared the effect of albendazole with ER stress-inducing cytostatic drug tunicamycin. Tunicamycin efficiently induced ER stress at the doses more than 50 ng/ml, evidenced by the increase of CHOP and ATF-4 (Figure 6(a)). However, tunicamycin did not induce apoptosis of SCC cells in terms of the cleavage of PARP-1 and caspase-3 (Figure 6(b)).

Since albendazole showed different potential on the induction of apoptosis between SCC cells and keratinocytes (Figure 4), we next determined the effect of albendazole on ER stress in both cells. Albendazole induced ER stress in SCC cells, while albendazole did not induce ER stress efficiently in keratinocytes even at 1.0 *μ*M concentration (Figure 6(c)).

### 3.3. Albendazole Decreases the Stemness of Cutaneous SCC Cells

**Figure 7:**
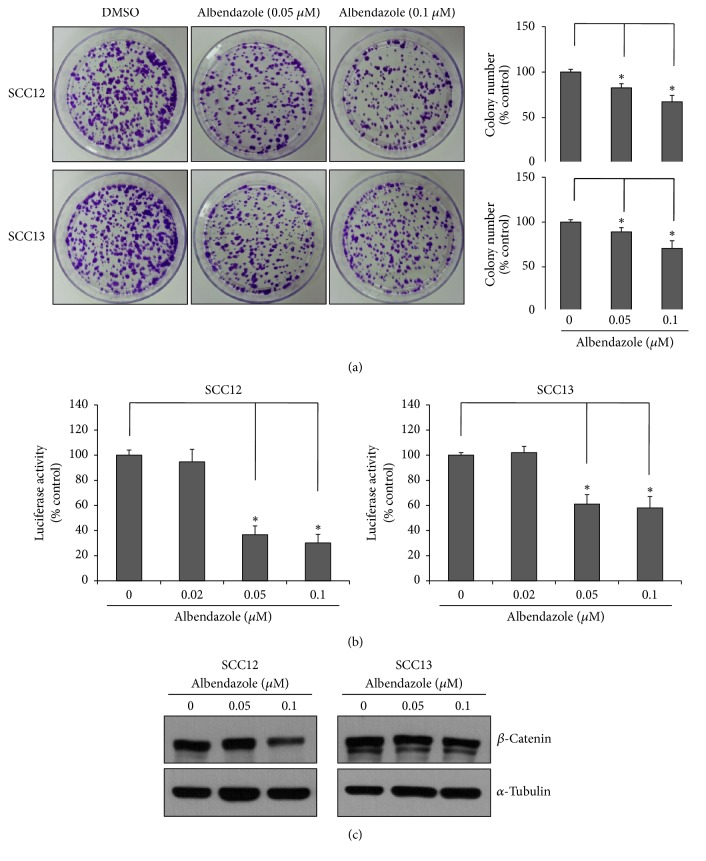
Effect of albendazole on cancer stemness. (a) SCC12 and SCC13 cells were treated with albendazole at the indicated concentrations for 24 h. Cells were then detached from culture dishes and then reseeded at a low concentration (3,000 cells per dish). Cells were cultured for 2 weeks and stained with crystal violet. Colony numbers were counted and plotted in right graph. Albendazole decreased the colony-forming ability of SCC cells. Data are expressed as percentage of control. The mean values ± SD are averages of triplicate measurements. *∗*P<0.01. (b) SCC12 and SCC13 cells were transduced with TOPflash reporter adenovirus and then treated with albendazole at the indicated concentrations for 24 h. The cell lysates were assayed for luciferase activities. Albendazole decreased the TOPflash activity at sublethal doses. Data are expressed as percentage of control. The mean values ± SD are averages of triplicate measurements. *∗*P<0.01. (c) SCC12 and SCC13 cells were treated with albendazole at the indicated concentrations for 24 h. The protein levels of *β*-catenin were detected by Western blot. Albendazole decreased the protein level of *β*-catenin. *α*-Tubulin was used for internal control.

The epidermal tumor maintenance relies on cancer stem cells, and Wnt/*β*-catenin signaling is essential for sustaining the cancer stemness [19]. Since albendazole decreased the cell viability of SCC cells at even low concentration such as 0.2 *μ*M (Figure 2), we speculated that albendazole can affect the stemness of cancer cells. To test this idea, we treated SCC12 and SCC13 cells with albendazole at nontoxic doses and performed colony-forming assay, an easy experimental method to determine stemness in culture condition [6]. As a result, albendazole significantly decreased the colony-forming ability of both SCC12 and SCC13 cells at nontoxic doses such as 0.05 *μ*M and 0.1 *μ*M (Figure 7(a)). Since Wnt/*β*-catenin signaling plays an important role in maintenance of cancer stemness, we examined the effect of albendazole on Wnt/*β*-catenin signaling. To this end, we used a well-established Wnt/*β*-catenin signaling reporter TOPflash system [20]. As expected, albendazole treatment resulted in decrease of TOPflash activity (Figure 7(b)). Consistent with these data, Western blot showed that protein level of *β*-catenin was decreased by albendazole treatment (Figure 7(c)). These data suggest that albendazole reduces the stemness of SCC cells via regulation of *β*-catenin signaling.

## 4. Discussion

As a common malignancy, the incidence of cutaneous SCC is increasing globally. Despite the several treatment modalities for cutaneous SCC, there is still a demand for novel pharmacotherapy that is more efficacious and safe. The repurposing/repositioning of drugs that have already been approved for other diseases is considered as an alternative way to develop new drugs, because time-consuming and expensive process can be avoided or shortened [21, 22]. In this study, we demonstrated that an antiparasitic drug albendazole has the potential to induce apoptosis of cutaneous SCC cells via triggering ER stress and reduces the stemness of SCC cells via regulation of *β*-catenin signaling.

ER is a membrane-bound organelle in eukaryotic cells, and it plays an important role in protein folding and maintaining cell homeostasis. Disruption of ER function by environmental and genetic factors can cause an accumulation of misfolded and/or unfolded proteins in the ER lumen, triggering unfolded protein response (UPR) and inducing ER stress [23]. In cases where ER stress cannot be reversed, eventually cells die by apoptotic process [16]. ER stress-induced apoptosis can be triggered via different pathways, including PKR-like ER resistant kinase (PERK), activating transcription factor-6 (ATF-6), inositol-requiring enzyme-1 (IRE-1), and caspase-12 [24]. In the present study, albendazole induced ER stress which was evidenced by increase of CHOP and ATF-4. CHOP is a transcription factor that positively promotes apoptosis following PERK activation [25]. ATF-4 is a transcription factor of PERK pathway, and it binds directly to an enhancer element called the amino acid response element (AARE) and upregulates transcription of CHOP [26]. In our study, albendazole also induced caspase-4 and caspase-12 in SCC cells. Interestingly, cells were pretreated with ER stress inhibitor 4-PBA; albendazole-induced cell death was significantly attenuated. Taken together, these data suggest that albendazole induces apoptosis by triggering ER stress.

The mechanism by which albendazole triggers the ER stress is not known. As previously recognized, albendazole has the inhibitory potential on microtubule formation [10]. Since microtubules serve their role as the track for biomaterials including proteins and nucleic acids, it can be speculated that inhibition of microtubule formation leads to disturbance of intracellular trafficking from ER to other organelles. This disturbance in ER homeostasis can create ER stress by activation of unfolded protein response (UPR) [27]. The precise mechanism underlying albendazole-induced ER stress remains to be clarified.

The canonical Wnt/*β*-catenin signaling pathway plays a central role in the carcinogenesis in several cancer types [28]. In addition, cancer stemness is strongly related to Wnt/*β*-catenin signaling pathway in epithelial cancer [19]. In this study, treatment with albendazole at nontoxic doses decreased the colony-forming ability of SCC cells, together with inhibition of Wnt/*β*-catenin signaling. These results suggest that albendazole can affect the SCC cells' behavior at multiple action points and strengthen its applicability as an antitumor drug. The relationship between ER stress and cancer stemness in SCC cells remains to be clarified. However, some studies in other systems give a hint for the correlation of ER stress and stemness of cancer cells. For example, excess ER stress markedly reduces the formation and maintenance of mammosphere which indicates the decrease of breast cancer stem cells. And the transcription factors from UPR and pluripotency pathways reciprocally influence each other in breast cancer [29]. Thus, there is a possibility that induction of ER stress by albendazole affects the intracellular signaling linked to stemness of cancer cells such as Wnt/*β*-catenin cascade. Elucidation of direct interplay between ER stress and Wnt/*β*-catenin signaling will be an interesting further study.

In summary, we demonstrate that albendazole has an antitumor effect on cutaneous SCC cells, providing the basis for extending the applicability of albendazole.

## Data Availability

The datasets generated and/or analyzed during the current study are available from the corresponding author upon reasonable request.
